# A comparison of the alterations of oral microbiome with fixed orthodontic therapy and clear aligners: a systematic review

**DOI:** 10.1080/20002297.2024.2372751

**Published:** 2025-02-01

**Authors:** Alessandra Lucchese, Marta Marcolina, Nicasio Mancini, Roberto Ferrarese, Serena Acconciaioco, Enrico Gherlone, Chiara Bonini, Maurizio Manuelli, Antonella Polimeni

**Affiliations:** a Department of Life Sciences, Health and Healthcare Professions, Link Campus University, Rome, Italy; b Unit of Orthodontics, School of Dentistry, Vita-Salute San Raffaele University, Milan, Italy; c Unit of Orthodontics, Division of Dentistry, IRCSS Ospedale San Raffaele Scientific Institute, Milan, Italy; d Unit of Dentistry, Research Center for Oral Pathology and Implantology, IRCCS Ospedale San Raffaele Scientific Institute, Milan, Italy; e Experimental Hematology Unit, Istituto di Ricovero e Cura a Carattere Scientifico (IRCCS) San Raffaele Scientific Institute, Milan, Italy; f Cell Therapy Immunomonitoring Laboratory Monitoraggio Immunologico Terapie Cellulari (MITiCi), Istituto di Ricovero e Cura a Carattere Scientifico (IRCCS) San Raffaele Scientific Institute, Milan, Italy; g Department of Medicine and Innovation Technology, University of Insubria (DIMIT), Varese, Italy; h Laboratory of Medical Microbiology and Virology University Hospital of Varese, Varese, Italy; i IRCCS Ospedale San Raffaele, Milan, Italy; j Private Practice Pavia, Bologna, Milan, Italy; k Department of Oral and Maxillo-Facial Sciences, Sapienza University of Rome, Rome, Italy

**Keywords:** Oral microbiome, clear aligners, orthodontic appliances, oral health, systematic review

## Abstract

**Aim:**

The oral microbiome plays a fundamental role in maintaining homeostasis of the oral cavity. In the last decade there has been an increasing use of clear aligners, which guarantee aesthetics and comfort for the patient. The aim of this work is to conduct a systematic review regarding the alterations in bacterial flora and oral health with aligner and fixed orthodontic therapy.

**Design:**

A systematic review was conducted following the PRISMA Statement. Using the search strategy “(clear aligners OR Invisalign) AND (fixed therapy OR fixed orthodont * therapy) NOT (thermoplastic retainers) AND (oral microbiome OR oral microbiota * OR oral microbiology * OR oral health)”, in the main scientific databases. Two scales were applied to assess the quality of scientific evidence: ROBINS-I and RoB 2.

**Results:**

A total of 484 articles emerged of which 9 met our inclusion/exclusion criteria. Afterwards the application of the rating scales, 1 article was found to be at low risk of bias, 6 at moderate and 2 at serious risk of bias.

**Conclusion:**

Both therapies cause an alteration of the oral microbiome, but the changes induced by the aligners seem to be compatible with a better oral health compared to fixed appliances.

## Introduction

The oral cavity microbiota counts over 700 species of bacteria, making it only the second, in terms of biodiversity, to that of the gut [1]. It is extremely varied and includes bacteria, fungi, viruses and protozoa. Oral microbiome plays a fundamental role in maintaining the homeostasis of the oral cavity and of systemic health too [2,3].

It is known that any biomaterial placed in an individual’s mouth leads to a dysbiosis; a perturbance of the balance of the resident flora which can promote the development of dental diseases [4]. For this reason, even orthodontic appliances, used to solve dental malocclusions, can induce an alteration of the oral microbiota, making the oral cavity more susceptible to pathological processes.

In the past years, many studies have been conducted to understand the impact of orthodontic appliances on oral microbiota, particularly regarding fixed orthodontic therapy. Fixed multibracket orthodontic appliances are used routinely in young and adult patients; however, they present certain drawbacks. Indeed, fixed appliances, mainly composed of a bracket which is bonded to the tooth surface, represent plaque-retention sites. This is due to the design of the metallic surface of the bracket, which represents an ideal ground for bacterial colonization and proliferation [4,5]. In addition, the greater difficulty for the patient to maintain good oral hygiene and the accumulation of subgingival plaque due to bone and periodontal remodeling induced by orthodontic forces result in a general increase in dental plaque during fixed orthodontic treatment. All of these factors lead to dysbiosis, with a consequent augmented risk for the patient to develop caries and periodontal disease, which represent the main side effects of fixed orthodontic therapy.

Due to the increasing request for orthodontic treatment in the adult population, in the past few years clear aligner therapy has become more and more popular. The therapy consists of multiple transparent tooth-shaped aligners which gently realign teeth. Aligners provide good comfort and aesthetics for the patient [6,7] and, since they are removable and present a less plaque-retentive design, guarantee better oral hygiene and periodontal health [8,9]. Indeed, at the end of orthodontic therapies, it seems that the various indices linked to the onset of white spots and periodontal indices: probing depth (PD), plaque index (PI), bleeding on probing (BOP), full mouth plaque score (FMPS) and full mouth bleeding score (FMBS) are significantly lower (*p* < 0.01) among patients with aligners compared to patients with fixed orthodontics [8–10].

The microbiological aspect of fixed orthodontic therapy has been well investigated, and multiple systematic reviews have confirmed that during the therapy a microbiological shift in the oral resident flora occurs with a predominance of gram-negative bacteria; these are associated with periodontal diseases such as *T. forsythia*, *P. gingivalis*, *P. intermedia* and *A. actinomycetemcomitans* [10–15]. Jung affirmed that fixed therapy also presented an increase in bacteria associated with caries, such as *S. mutans, S. salivarius* and *Lactobacilli* [16].

Although numerous studies and literature reviews [8,9,17–19] have researched the oral health conditions of patients treated with aligner therapy, highlighting better periodontal and cariologic conditions compared to those treated with fixed therapy, only a few studies have evaluated the microbiological aspect of this kind of therapy and no literature review is present. Since the analysis of microbiota is fundamental to achieving a real understanding of clinical oral health conditions, the purpose of the present study is to conduct a systematic review of the articles published in the last 10 years in order to address the following questions:

- Do clear aligners cause an alteration of oral microbiota?
- Do clear aligners and fixed therapy have the same effect on oral microbiota?

## Materials and methods

### Protocol and registration

The present systematic review followed the PRISMA 2020 guidelines [20] (Preferred Reporting Items for Systematic Reviews and Meta-Analyses). No funding was provided for the realization of the present review. This systematic review is registered on the PROSPERO database with the following registration number: CRD42023426054.

### Eligibility criteria

Inclusion and exclusion criteria for the studies included in the revision process were defined before the study, based on the PICOS (Participants, Intervention, Comparison, Outcome, Study) (Table 1).

**Table 1.** Inclusion and exclusion criteria.

Inclusion criteria	Exclusion criteria
RCTs, nRCTs, cohort studies At least 2 time points for analysis (with at least one before the beginning of treatment) At least 10 patients analyzed Trials analyzing patients treated with clear aligners or comparison between aligners and fixed therapy Trials analyzing patients with good systemic health, who were not taking medications	Case reports, review articles, systematic reviews, thesis and author opinion In vitro studies Studies that included patients treated with dental extractions

### Information sources and search strategy

An electronic search of the keywords (clear aligners OR Invisalign) AND (fixed therapy OR fixed orthodont* therapy) NOT (thermoplastic retainers) AND (oral microbiota* OR oral microbiology* OR oral health) was performed in the electronic databases PubMed, Cochrane Library, Embase, Web of Science, Scopus, Ovid, Dentistry and Oral Sciences Source and also Gray literature was investigated on OpenGray (www.opengrey.eu) until 30 May 2023. A manual search was conducted in the library at Vita Salute San Raffaele University and in the references of the eligible studies, in order to find additional articles. No place, language or publication date restrictions were utilized.

### Study selection

The selection of the studies was processed according to PRISMA guidelines [20] and consisted of two phases. An initial screening of articles based on title and abstract, following the research question defined by the PICOS and inclusion and exclusion criteria, was performed independently by two reviewers (A.L., M.M.), who to minimize bias had experience in Oral Microbiology. To ensure consistency, 15 articles were independently screened by the two reviewers, and the concordance between both was measured using the Kappa index. In the second phase, full-text articles were recovered from a pool of potentially eligible studies. To avoid missing relevant studies, reference lists of full-text papers were manually searched by the two reviewers, and articles found in this way were eventually added to the pool of full-text articles to be evaluated. Discussions were held to resolve any disagreements; when a resolution could not be found, a third reviewer was consulted (N.M.), and if data were not clear enough, an attempt was made to contact the authors of the analyzed studies by email. The latter studies pool was later assessed for eligibility based on responses obtained from the authors of individual articles contacted by email.

### Data collection and analysis

The data extraction of the included studies was performed by the two reviewers (A.L., M.M.) following a discussion with the third reviewer (N.M.) to define the variables to be extracted and to solve disagreements in data extraction. A customized table was realized using Microsoft Excel® 2021 and used for data extraction, as shown in Table 2.

**Table 2.** Characteristics of studies included in the review.

Author/year	Study design	Sample size (F/M)	Appliance analyzed	Sample collection time	Collection method	Microbial analysis	Microbiological outcome
Wang (2020)	Controlled clinical trial	15	Fixed (G1) and Invisalign® (G2)	At 6 months from treatment start	Saliva sample	16S rNA gene sequencing	↑ F, fa in G1; ↑ TM7, N in G2
Guo (2018)	Prospective cohort study	10 (10/0)	Clear aligners	T0, T1, T3	Subgingival plaque	16S rNA gene sequencing	T2: ↑ R, A
Zhao (2020)	Prospective cohort study	25 (22/3)	Invisalign®	T0, T1 (6 months later)	Saliva sample	16S rNA gene sequencing	T2: ↑ Ba; ↓ P, Pp
Lombardo (2021)	Prospective cohort study	27	Fixed (G1) and clear aligners (G2)	T0, T1, T2	Subgingival plaque	PCR	G1: ↑ Fn
Karkhaneci (2013)	Prospective cohort study	42	Fixed (G1) and Invisalign® (G2)	T0, T1, T2, T3	Subgingival plaque	BANA test (OraTec Corp, Manassas, Va)	G1: ↑ BANA score
Sifakakis (2018)	Prospective cohort study	30	Fixed self-ligating (G1) and clear aligners (G2)	T0, T1, T2	Saliva sample	PCR	G1: ↑ Ssa
Levrini (2018)	RCT	77 (52/25)	Fixed (G1) and Invisalign® (G2)	T0, T1 T2	Subgingival plaque	PCR	G1: ↑ biofilm mass
Shokeen (2022)	Prospective cohort study	24 (8/16)	Fixed (G1) and clear aligners (G2)	T0, T1, T2, T3, T4	Supragingival plaque	16S rNA gene sequencing	G1: ↑ Le, Se, P, V, C TM7; ↓ R, La, H
Song (2023)	Prospective cohort study	55	Clear aligners (G1		Saliva sample	16S rNA gene sequencing	↑ Su, R, Lach in WSL group

V: *Veillonella;* S: *Streptococcus;* P: *Prevotella;* H: *Haemophilus;* Po: *Porphyromonas;* Se: *Selenomonas;* R: *Rothia;* F: *Fusobacterium;* Pi: *Prevotella intermedia;* Aa: *Aggregatibacter actinomycetemcomitans;* Co: *Corynebacterium*; Ca: *Capnocytophaga;* La: *Lautropia*; Cr: *Campylobacter rectus;* Fn: *Fusobacterium nucleatum*; Td: *Treponema denticola;* My: *Mycoplasma*; Be: *Bergeyella;* Ba: Bacillus; Pg*: Porphyromonas gingivalis;* A: Actynomices; Le: *Leptorichia;* Sm: *Streptococcus mutans*; Ss: *Streptococcus sobrinus;* Pp: *Prevotella_pallens_ATCC_700 821;* Ag: *Actinomyces_graevenitz ii_F0530;* Lepto: *Leptotrichia_sp.oral_clone_EI 013;* Sp: *Streptococcus_parasanguinis_FW213;* Lepto36: *Leptotrichia_sp.oral_clone_ FP036;* Tf: *Tannerella forsythia;* Ssa: *Streptococcus sanguinis*; Lba: *Lactobacillus acidophilus;* Su*: Subdoligranulum;* Lach*: Lachnoanaerobaculum* Nd: *not detected;* ÷: compared to; ssd: statistically significant difference; fa: functional activity.

The data to be extracted were first author and year of publication, study design, sample size, number of comparison groups, average patient age, sample collection time, microbial analysis method, bacteria analyzed and microbiological outcome.

### Risk of bias

The risk of bias was assessed using the ROBINS-I (Risk Of Bias In Non-Randomized Studies of Intervention) scale for non-randomized studies [21] and RoB 2 [22] (Revised Cochrane risk-of-bias tool for randomized trials) for RCTs. The following biases were analyzed for each nRCT study: confounding bias (D1), selection bias (D2), bias in classification of interventions (D3), bias due to deviation of intended interventions (D4), bias due to missing data (D5), bias in measurement of outcomes (D6) and bias in the selection of reported result (D7).

For randomized trials, the biases evaluated were bias of randomization process (d1), bias due to deviations from intended interventions (d2), bias due to missing data (d3), bias in measurement of outcomes (d4) and bias in selection of reported result (d5).

After the evaluation of every domain, the authors attributed to all studies an overall risk of bias, which was ‘low’, ‘moderate’, ‘serious’ and ‘critical risk of bias’ for non-randomized trials and ‘low risk’, ‘some concerns’ and ‘high risk of bias’ for RCTs. Risk of bias of included studies were exposed graphically with the risk of bias traffic light plot of ROBINS-I (Figure 2) and Rob2 (Figure 3) assessment using robvis [23]. With the same software, a weighted bar plot was realized to summarize the outcome level of the review. Kappa statistics were used to estimate the inter-rater reliability between the two independent reviewers (A.L., M.M.) to assess the risk of bias of the articles.

## Results

### Study selection

**Figure 1.**
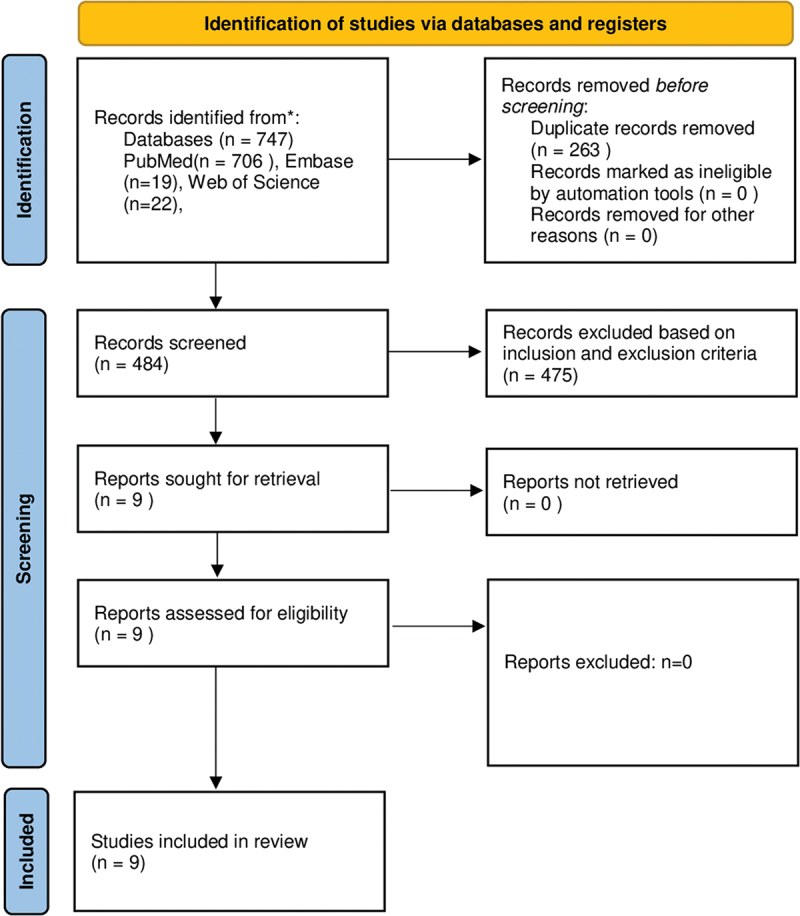
PRISMA 2020 flowchart of selection process.

Database searches retrieved 747 records, from which duplicates were removed, leaving 484 articles to screen. Titles and abstracts of these papers were screened by two reviewers (A.L., M.M.), and a good agreement between them was reported (Kappa, 0.85). Any disagreements were resolved by the third reviewer (N.M.). Based on exclusion criteria, 475 articles were rejected, resulting in nine papers that were integrally read to assess eligibility. All these studies met the inclusion criteria and were included in the review. The full PRISMA 2020 statement flowchart is displayed in Figure 1, and the data synthesis of all the articles analyzed is reported in Table 2.

### Study characteristics

#### Type of study

Of the nine studies included in the revision following the selection process, eight [24–31] were non-randomized trials and one RCT [32].

#### Characteristics of the participants

The included studies presented at least two groups of participants, one with fixed orthodontic appliances and one with aligners. The only exceptions were the studies by Guo [27] and Zhao [25] and Song [31], who conducted the study on a unique population of clear aligner patients. This significantly influenced the total score of the studies. Every study excluded patients with present or remote history of periodontitis, with active caries or patients who were currently, or in the last 3 months, under antibiotic therapy.

#### Characteristics of microbiologic analysis

Microbiological samples were collected from subgingival plaque in four studies, from supragingival plaque in one study and from saliva in four of them. For this type of analysis, five studies used innovative NGS method based on the sequencing of 16S RNA; three studies used the common PCR analysis and only one study conducted the analysis with the BANA test, a rapid side-chair test usually used to detect anaerobic bacteria involved in periodontitis.

### Quality assessment

A careful point-by-point evaluation of the domains of the rating scales has highlighted that there are numerous lacks of detail in many of the articles analyzed [25–27,32], especially regarding selection bias, bias due to deviation of intended interventions and bias in the selection of reported result.

We performed a scrupulous analysis of the studies included in the review and summarized the main methodological issues in this section.

The ROBINS-I scale was used to assess quality in non-randomized trials. The articles of Guo [27] and Zhao [25] presented a high risk of bias in D1 caused by the absence of a control group. Nevertheless, the study of Zhao [25] seems to have been subjected to a funding bias due to the support of ‘Invisalign® Awards’. Among the analyzed studies, only that by Lombardo *et al*. achieved a low risk of bias in D1, since the allocation of patients to one or another group of intervention was not based on the orthodontist’s personal choice, but on the IOTN index. Due to the detailed description of the two groups, this study was the only one to achieve a low risk of bias, even in D3. The study of Wang *et al*. [24] obtained a moderate risk of bias in D2 since they performed an evaluation only on a small subgroup of the population involved in the study. The study of Song *et al*. [31] presented a critical judgement in D3 due to a lack of description of the classification of the interventions; even the timing of sample collection was not well described. The study conducted by Kharkhaneci *et al*. [28], which was the oldest, was subjected to a moderate risk of bias in D6 due to the use, as a method of microbiological evaluation, of the BANA test, which presents a low specificity. The only RCT included in the review [32] was analyzed with the Rob2 scale. The paper obtained a low risk of bias in every domain, excepted for D1, since the randomized allocation sequence was not well described. Moreover, it was not a blinded study, since both the operator and the patients were aware of the intervention assigned or received.

To obtain an immediate visualization of the quality of each study and, in general, of the revision, the visualization tool robvis (risk of bias visualization) was used. Table 3, realized in Microsoft Excel® 2021 as the software requested, was imported into the visualization tool, and two graphic plots were generated. The items of the table were as follows:

- Study: a number identify each study included in the review
- Domain from 1 to 5/7, according to the type of study analyzed
- Overall: the global judgment of the studies, as an outcome of the quality assessment evaluation

**Table 3.** Summary table of judgement assigned to each domain evaluated for all the studies.

Study	D1	D2	D3	D4	D5	D6	D7	Overall
*Study 1* (Wang)	Moderate	Moderate	Moderate	Low	Low	Moderate	Moderate	Moderate
*Study* 2 (Guo)	Serious	Low	Moderate	Low	Low	Moderate	Low	Serious
*Study 3* (Zhao)	Serious	Moderate	Moderate	Low	Low	Moderate	Low	Serious
*Study* 4 (Lombardo)	Low	Low	Low	Low	Moderate	Low	Low	Low
*Study* 5 (Karkhaneci)	Moderate	Low	Low	Low	Moderate	Moderate	Low	Moderate
*Study* 6 (Sifakakis)	Moderate	Low	Low	Moderate	Low	Moderate	Low	Moderate
*Study* 7 (Levrini)	Some concerns	Low	Low	Low	Low	/	/	Some concerns

**Figure 2.**
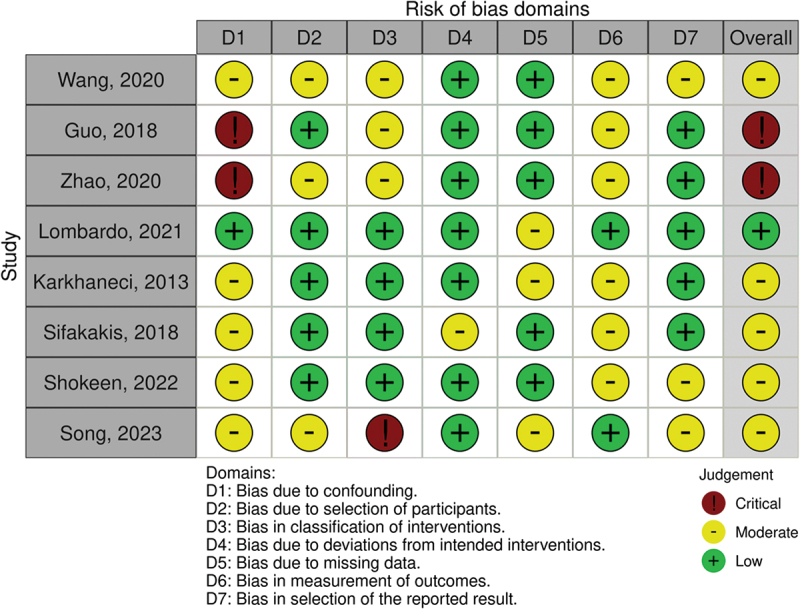
Traffic light plot for nRcts.

**Figure 3.**
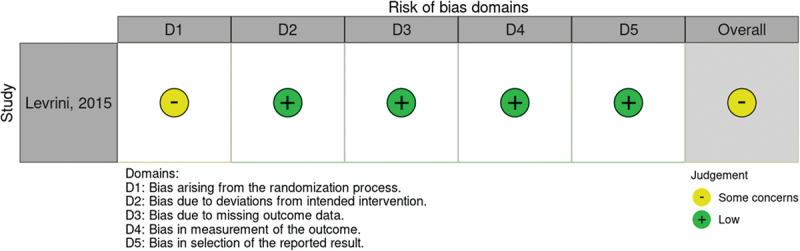
Traffic light plot for RCTs.

**Figure 4.**
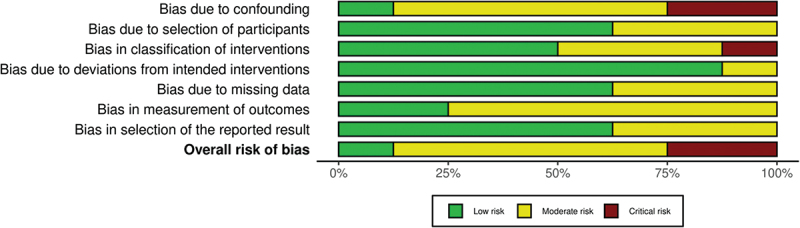
Weighted bar plot for the overall risk of bias of the review.

A ‘traffic light’ plot is shown in Figure 2 for non-randomized trials and, in Figure 3, for RCTs. Furthermore, with the same software, a weighted bar plot (Figure 4) was created. It provided support in the statement of the total quality of the present review, which was declared as at overall moderate risk of bias.

### Data synthesis

We grouped the studies by the type of orthodontic appliance analyzed, the sample collection methods, the microbiological analysis methods and study design for each group. A subgroup analysis by age was not performed, since the age of the population was similar in the seven studies we evaluated.

## Discussion

An imbalance of the oral microbiota could lead to an alteration of the oral health status with an increased risk of enamel demineralization, caries, gingivitis and periodontitis. Since one of the main factors which induces an imbalance of the oral resident flora is the positioning of external appliances in the oral cavity, literature has constantly investigated the link between orthodontic therapy and oral microbiological changes. The residence time in the oral cavity and the design of the appliance, in terms of plaque-retentive surfaces, are two of the principal determinants of microbiological shifts during orthodontic therapy [33].

Fixed therapy, which remains 24/7 in the patient’s mouth and comports a high level of plaque accumulation, causes an increase of gram-negative bacteria, usually associated with periodontitis, such as *T. forsythia*, *P. gingivalis*, *P. intermedia*, *T. intermedia* and *A. actinomycetemcomitans*. Literature has also focused on the increased risk of white spot lesions and caries during orthodontic multibracket therapy, founding augmented levels of *S. mutans, S. salivarius* and Lactobacilli 3 months from the beginning of therapy [33–36].

However, times change, and in the last decade, clear aligners have reached more and more popularity, especially in the adult population, given their comfort and aesthetics. At the same time, there is a lack of studies which have investigated microbiological changes after this kind of therapy, their impact on oral health and the differences with fixed therapy.

Following the analysis of the seven studies included in this review, we can state that clear aligners cause, at least in the short term, a dysbiosis of the oral resident flora, as demonstrated by the studies of Guo [27] and Wang [24]. This agrees with Øilo *et al*. [4], which stated that every external disposable placed in the oral cavity causes a microbiological perturbation. However, Guo *et al*. [27] and Zhao et al. [25] evaluated the alpha diversity index and did not find a reduced diversity in the microbial community after clear aligner therapy. A rich and heterogeneous flora is often representative of a healthy and stable microbiological community, in contrast to a population dominated by few species and more susceptible to a pathogenetic status [37,38]. Lombardo *et al*. [26], evaluating the TBL (Total Bacterial Load) in patients treated with fixed and clear aligner therapy, found in the former a major increase of TBL in respect of the aligner group. These results are even confirmed by Levrini et al. [32], who found a minor biofilm accumulation at 3 months from the beginning of therapy in patients treated with clear aligners compared to those treated with fixed therapy. The most plausible hypothesis behind these findings is that clear aligners can be removed by the patient, allowing better oral hygiene with minor plaque accumulation.

Concerning the alteration of levels of periodontal species during aligner therapy, results from the studies of Guo et al. [27] and Kharkhaneci et al. [28] show that the levels of *A. actynomicetemcomitans*, *P. intermedia*, *C. rectus, F. nucleatum, T. denticola, P. gingivalis* and *T. Forsythia* were comparable to those registered before therapy. However, both studies presented a moderate risk of bias due, in the first case [27] to a limited follow-up period of 3 months; in the second [28] to the use of an unspecific microbiological analysis (BANA test) [39]. In fact, the study of Lombardo [26], which analyzes the dynamics of periodontopathogen species in clear aligner patients after 6 months of therapy, agrees only in part with the results of the previous studies. *T. denticola, P. gingivalis and T. forsythia* presented the same levels before and at 6 months of therapy in patients treated with either clear aligners or fixed therapy. Only the levels of *F. nucleatum*, a bacterium of the orange complex of Socransky, were significantly increased in the fixed group. Even if it is not simple to make an association between microbiological and clinical findings, it seems that the microbiological results from these studies find a clinical proof in papers and systematic reviews [8,9,17,32,40] which demonstrated a minor alteration of periodontal index (BoP, IP, PD) in patients treated with clear aligners compared to those treated with fixed therapy, as confirmed by Shokeen *et al*. [30].

In the present review, the study which evaluated the levels of caries-related species [25], such as *S. sobrinus, S. salivarius, S. sanguinis*, found that they were stable compared to those registered before the beginning of therapy. In contrast, Wang et al. [24] and Sifakakis et al. [29] found a statistically significant increase of *S. sanguinis* and *Firmicutes* genera (of which *S. mutans* and Lactobacillus spp are part) in the group of patients treated with fixed therapy. These results may lead to the hypothesis of a minor risk of white-spot lesions and caries, one of the major side effects of fixed therapy, in clear aligner treatment [41]. This had already been confirmed by a previous study [42] which evaluated, through quantitative light-induced fluorescence, the number of white spot lesions in patients treated with fixed and clear aligner therapy. They found the highest number of white spot lesions in patients treated with multibracket therapy. However, there was a slight difference between the extension of lesions in the two groups of patients: in the fixed group, there were punctiform lesions, localized prevalently at the sides of brackets (where dental plaque accumulated more); otherwise, in the aligner group, lesions covered a more extensive area. This could be explained by the way that aligners cover teeth surfaces for a long period, reducing their exposition to salivary flow with less possibility to explicate its buffer and remineralization potential [43,44]. This association was also confirmed by the study of Song et al. [31], which stated that an increase in WSL in patients treated with clear aligners correlated with augmented levels of cariogenic bacteria due to a reduced exposure of teeth surfaces to salivary flow.

### Limitations

Despite the present review benefiting from moderate scientific quality, it is subject to some limitations. First, only a qualitative analysis was conducted, since the heterogeneity of the procedures used for microbiological analysis did not allow a meta-analysis of the studies included. Furthermore, all the studies investigated presented a lack of description of the appliances involved in the studies. For example, none of the studies cited the presence or not of attachments, which could constitute a plaque-retention factor. Lastly, in the included studies, there was no long-term follow-up.

## Conclusions

The previous findings lead us to the following conclusions:

- Both fixed and aligner therapy cause dysbiosis: an alteration of oral microbiota
- Microbiological alteration due to aligner therapy is more compatible with good oral health status
- In the next few years, it is necessary that studies aim to have a longer follow-up to evaluate long-term changes in the oral flora in patients treated with clear aligners.

## Key points

- Both fixed orthodontic therapy and clear aligners cause an alteration of oral microbiota.
- The microbiological changes related to clear aligners are more compatible with a state of oral health.

## Data Availability

The datasets used and/or analyzed during the current study are available from the corresponding author on reasonable request.
